# Suicide risk, personality disorder and hospital admission after assessment by psychiatric emergency services

**DOI:** 10.1186/s12888-019-2145-0

**Published:** 2019-05-23

**Authors:** Mark Van Veen, André I. Wierdsma, Christine van Boeijen, Jack Dekker, Jeroen Zoeteman, Bauke Koekkoek, Cornelis L. Mulder

**Affiliations:** 1 Research Group for Social Psychiatry and Mental Health Nursing, University of Applied Science, Nijmegen, The Netherlands; 2 Altrecht Mental Health Services, Utrecht, The Netherlands; 3 Epidemiological and Social Psychiatric Research Institute, Department of Psychiatry, Erasmus MC, Rotterdam, The Netherlands; 4 GGNet Mental Health Services, Apeldoorn, The Netherlands; 5 Faculty of Behavioural and Movement Sciences, VU Faculties, Amsterdam, The Netherlands; 6 Arkin Mental Health Care, Department of Emergency Psychiatry, Amsterdam, The Netherlands; 7 Pro Persona Mental Health Services, Wolfheze, The Netherlands; 8 Parnassia Psychiatric Institute, Rotterdam, The Netherlands

**Keywords:** Suicide risk, Personality disorder, Voluntary psychiatric admission, Involuntary admission psychiatric emergency service

## Abstract

**Background:**

The main objectives of the mobile Psychiatric Emergency Services (PES) in the Netherlands are to assess the presence of a mental disorder, to estimate risk to self or others, and to initiate continuity of care, including psychiatric hospital admission. The aim of this study was to assess the associations between the level of suicidality and risk of voluntary or involuntary admission in patients with and without a personality disorder who were presented to mobile PES.

**Methods:**

Observational data were obtained in three areas of the Netherlands from 2007 to 2016. In total, we included 71,707 contacts of patients aged 18 to 65 years. The outcome variable was voluntary or involuntary psychiatric admission. Suicide risk and personality disorder were assessed by PES-clinicians. Multivariable regression analysis was used to explore associations between suicide risk, personality disorder, and voluntary or involuntary admission.

**Results:**

Independently of the level of suicide risk, suicidal patients diagnosed with personality disorder were less likely to be admitted voluntarily than those without such a diagnosis (admission rate .37 versus .46 respectively). However, when the level of suicide risk was moderate or high, those with a personality disorder who were admitted involuntarily had the same probability of involuntary admission as those without such a disorder.

**Conclusions:**

While the probability of voluntary admission was lower in those diagnosed with a personality disorder, independent of the level of suicidality, the probability of involuntary admission was only lower in those whose risk of suicide was low. Future longitudinal studies should investigate the associations between (involuntary) admission and course of suicidality in personality disorder.

## Background

While suicide rates vary considerably between nations and over time, ranging from 3.9 suicides per 100,0000 people in the Eastern Mediterranean to 13.2 in South East Asia, rates of attempted suicide are relatively similar over a wide area [1]. In the Netherlands, injuries caused by suicide attempts led to 93.8 treatments at emergency departments and 56.3 general hospital admissions per 100,000 inhabitants in 2015 [2]. Many people who report suicidal thoughts or attempt suicide are seen by Psychiatric Emergency Services (PES), whose main objectives are to assess the presence of a mental disorder, to estimate risk to self or others, and to initiate an intervention (including psychiatric hospital admission). Following attempted suicide, PES often are called upon by general hospital emergency services. In the Netherlands, more often General Practitioners (GP’s), ambulance services and the police ask PES for an assessment. Assessing suicide risk is therefore a core task of PES: in the Netherlands, mostly done by a community psychiatric nurse and a psychiatrist. 30% of a total 150,000–175,000 assessments each year, are related to suicidal behaviour [3]. Some 16% of all patients assessed within office hours are admitted to a psychiatric hospital, and some 28% of those assessed outside office hours. However, regional differences apply (range 5–35%) [4, 5].

### Suicide risk, personality disorders and admission

When the perceived risk of suicide is high, PES may initiate psychiatric hospital admission, either with or without the patient’s consent. It is a matter of professional debate whether or not suicidal patients should be admitted: some argue that protection should have the greatest priority [6], while others contend that restricting a patient’s autonomy may increase the risk of suicide during and after admission [7, 8]. Importantly, admission cannot prevent suicide [9]. Interestingly, two studies found no association between suicide risk and hospital admission in the Netherlands [10, 11], others found that the probability of involuntary admission was increased by suicide risk in Israel [12], the USA [13, 14] and Germany [15].

To date, however, we have found no studies that investigated the interactions between suicidality, admission, and the type of mental disorder. In the absence of empirical evidence, clinical experience suggests that the rate of admission is higher in patients in whom acute suicidality is related to factors such as depression or psychosis than it is in those in whom it is related to having a personality disorder.

While clinicians vary substantially in the ways they perceive suicide risk in patients with a personality disorder (most often a borderline personality disorder), it is unknown whether voluntary or involuntary admission is effective in reducing the level of suicidality. In some cases the level of suicidality may even increase, especially in patients with regressive behaviours, e.g. resulting in physical aggression towards self and others [16–19]. As three qualitative studies have shown, the hospitalisation of chronically suicidal patients may become repetitive, and may intensify suicidal behaviour [17, 20, 21]. Since there are no prospective studies, it is difficult to judge when it is justified to admit a suicidal patient with a personality disorder. Some patients with a personality disorder may get into conflict with staff and other patients during admission, particularly in the case of involuntary admission, resulting in a negative chain of events in which suicidal behaviour, aggression and self-harm increase [20–23].

### Aims of the study

The aim of this study is to assess the association between level of suicide risk, a diagnosis of personality disorder, and risk of voluntary or involuntary admission by the PES. We hypothesized that suicidal patients with a personality disorder have a lower probability of admission.

## Methods

### Study design

In this observational study we used data from an electronic patient file designed specifically for use in PES, i.e. a web-based clinical support system comprising information on sociodemographic variables, psychiatric symptoms, psychiatric diagnoses and environmental data. We selected data over a ten-year period (2007–2016) of all patients aged between 18 and 65 seen by mental-health services in the two largest cities in the Netherlands (Amsterdam and Rotterdam) and in one midsize city (Apeldoorn) in a more rural area.

Patients were seen by the PES (a psychiatrist together with a nurse, or a medical doctor or resident in psychiatry, supervised by a psychiatrist) on request of others: usually the general practitioner, but sometimes also on request of the police or an emergency department of a general hospital.

### Data collection

#### Sociodemographic variables

We collected data regarding gender, ethnicity (born in the Netherlands vs. born in another country), ethnicity and age.

#### Clinical factors

Clinical factors, including level of suicide risk, were assessed using the Severity of Psychiatric Illness scale (SPI). The SPI was originally developed as a patient-level decision support tool to assess the need for services [24]. It contains 14 items, including level of suicide risk, substance abuse, and danger to others. While two studies [24, 25] have used the SPI on an item level rather than a total-score level, we focused on four items that were previously found to be associated with risk of admission [25]: level of suicide risk, level of substance abuse, danger to others, and motivation for treatment. Each item was rated on a 4-point scale from 0 to 3, with 0 indicating no risk and 3 indicating a high risk. The SPI is considered reliable(24)The Dutch version of the SPI had an overall inter-rater reliability of kappa 0.76 [25].

#### Psychiatric diagnoses

Clinicians either based their DSM-IV diagnoses on a clinical interview, or adopted the diagnoses from the psychiatric files. These diagnoses were registered in broad categories such as ‘psychotic disorder’, ‘depressive disorder’ or ‘personality disorder’. The category ‘other’ contained diagnoses such as anxiety disorder or PTSD. Clinicians also registered different subtypes of personality disorder. For the analyses, we grouped the subtypes of personality disorders together, as no structured interview for assessment of a personality disorder was performed, and therefore the reliability of assessing subtypes of personality disorders in the context of the PES can be questioned. Clinicians could register more than one diagnosis. When personality disorder was registered as one of the diagnoses, this patient was coded as having a personality disorder, beside possible other (axis I or axis II) diagnoses.

#### Environmental factors

Family requests for admission were assessed separately on the basis of a dichotomous item asking whether or not the family had requested admission. The level of family involvement was assessed on the basis of one item of the SPI, which was also rated on a 4-point scale from 0 to 3, with 0 indicating significant family involvement and 3 indicating absence of family involvement.

#### Outcome measure

Our outcome measure was admission to a psychiatric hospital through the PES, either voluntarily or involuntarily. The four criteria for emergency involuntary admission in the Netherlands are [1] the presence of a mental disorder (this is not specified in Dutch Mental health law, but in practice it is mainly a psychotic, bipolar I, or severe depressive disorder), [2] causing danger to self or others, [3] the lack of an alternative way of averting the danger and [4] unwillingness to be hospitalised.

### Statistical analysis

Descriptive statistics were used to summarize the data on socio-demographic characteristics, clinical factors, diagnoses, and admission (Table 1). Logistic regression analysis was performed to assess the impact of personality disorder,on the association between the level of suicidality and the likelihood of admission, while controlling for gender, age, and danger to others (Table 2). Model comparison was based on the Akaike Information Criterion (AIC). To assess the fit of the final models, we calculated the Hosmer-Lemeshow goodness-of-fit statistic and the area under the receiver operating characteristic (ROC) curve [26].

**Table 1:** Admission patterns and characteristics in patients assessed by the Psychiatric Emergency Services

		Total no. of patients assessed *N* = 71,707 (100%)	No admission *N* = 42,572 (59%)	Voluntary admission *N* = 14,346 (20%)	Involuntary admission *N* = 14,789 (21%)
Sociodemographic variables					
Gender	Male	55.1	57.4	19.6	23.0
	Female	44.9	61.7	20.6	17.7
Ethnicity	Dutch	67.3	59.1	21.1	19.8
	Other	8.2	54.9	17.9	27.2
	Unknown	24.5	61.6	17.6	20.8
Age	18–38	49.5	59.9	18.2	21.9
	39–59	44.4	58.8	21.7	19.5
	60–65	6.1	59.5	22.6	17.9
Clinical factors					
Suicide risk	None (0)	35.4	62.0	15.9	22.1
	Low (1)	40.3	63.5	17.7	18.8
	Moderate (2)	16.3	55.3	28.4	16.3
	High (3)	8.0	35.2	32.9	31.9
Substance abuse	None (0)	54.0	62.5	20.1	17.4
	Low (1)	13.2	61.8	18.7	19.5
	Moderate (2)	16.1	56.7	17.9	25.4
	High (3)	16.7	50.1	22.7	27.2
Danger to others	None (0)	54.3	72.1	21.9	5.9
	Low (1)	30.3	56.5	20.7	22.8
	Moderate (2)	8.5	25.9	15.4	58.7
	High (3)	7.0	13.1	7.4	79.4
Motivation for treatment	None (0)	25.4	75.7	22.9	1.3
	Low (1)	31.2	64.4	30.8	4.8
	Moderate (2)	22.2	54.8	17.4	27.8
	High (3)	21.2	37.1	3.5	59.4
Psychiatric diagnoses					
Diagnosis axis I DSM-IV	Depressive disorder	12.6	63.6	28.7	7.7
	Psychotic disorder	32.1	38.8	20.3	40.9
	Other	55.3	68.9	20.0	11.1
Diagnosis axis II DSM IV	Personality disorder^a^	16.2	62.8	23.0	14.2
Environmental factors					
Admission requested by family	Not applicable	58.3	66.3	15.9	17.8
	Yes	28.0	28.3	36.6	35.1
	No	13.7	93.3	3.7	3.1
Family involvement	None (0)	44.1	61.4	21.0	17.6
	Low (1)	21.4	60.2	19.9	19.9
	Moderate (2)	15.7	58.5	20.0	21.6
	High (3)	18.8	54.4	17.8	27.9

^a^11 subtypes of personality disorder (according to DSM-IV) grouped together

**Table 2:** Probability of voluntary or involuntary admission in patients with suicide risk and personality disorder

	Voluntary admission B (SE)	Exp (B)	Involuntary admission B (SE)	Exp (B)
Intercept ^a^	−1.395 (.020)		−1.158 (.033)	
Suicide risk	0.398 (.013)	1.49	−0.037 (.018)	0.96
Personality disorder	−0.508 (.049)	0.60	−0.929 (.080)	0.39
Interaction effect	0.142 (.031)	1.15	0.307 (.045)	1.36
AIC ^b^	−32.3		−63.3	
AUC	.72		0.80	

^a^Controlling for age (grand-mean centered), gender (effect-coded), and danger to others

^b^AIC in smaller-is-better-form, comparing models with and without interaction effect

To explore differences in outcomes when alternative strategies were used, we performed sensitivity analyses. Since risk assessments were grouped within clinicians and service organizations, generalized mixed models were fitted to determine the impact of the hierarchical structure of the data. Next, to control for the fact that the absence of suicidality does not automatically mean that the patient will not be admitted, we explored three approaches other than controlling for danger to others. First, we defined alternative suicide-risk categories; secondly, we split the file into ‘no danger to others’ and ‘low to high danger to others’; and thirdly we restricted suicide risk by excluding patients with no suicide risk and patients with a moderate suicide-risk but a high risk of danger to others. As these approaches produced no relevant differences, we only report models controlling for danger to others. A full account of the sensitivity analyses is available on request from the second author. All statistical analyses were performed using SPSS version 24.0 (SPSS Inc., Chicago, IL).

## Results

A grand total of 71,707 patients were assessed between 2007 and 2016, of which nearly 70% had been born in the Netherlands. Nearly 30% of the referrals had been made by GPs; in almost 40% of these cases, suicidality had been the reason for referral. Over half of the assessed patients (54.6%) were suicidal at the time of referral, with at least a moderate or high score on the SPI item. In terms of their diagnoses, over 30% had a psychotic disorder and over 16.2% had a personality disorder, mostly a borderline personality disorder (7.2%), followed by an otherwise unspecified personality disorder (6.4%), and an anti-social personality disorder (1.4%). For all characteristics, see Table 1.

The voluntary admission rate was 17.7% for patients with a low suicide risk, 28.4% for patients with a moderate risk, and 32.9% for patients with a high risk. The involuntary admission rate was 18.8% for patients with a low suicide risk, 16.3% for patients with a moderate risk and 31.9% for patients with a high risk.

**Fig. 1:**
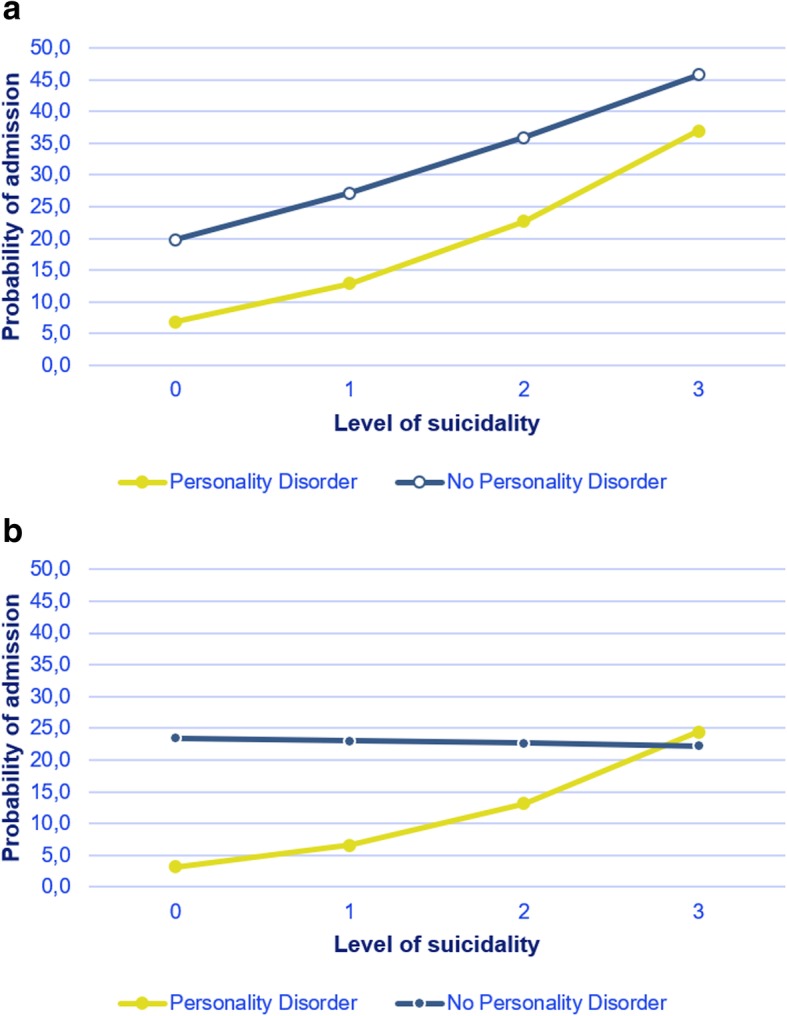
**a** Voluntary admission by suicide risk and personality disorder (or no personality disorder) at fixed values for age gender and danger to others. **b** Involuntary admission by suicide risk and personality disorder (or no personality disorder) at fixed values for age, gender, and danger to others

Table 2 shows that the probability of voluntary admission to a psychiatric hospital for patients with a specific level of suicide risk was affected by the presence of personality disorder. Overall, patients diagnosed with personality disorder were less likely to be admitted than other patients. For patients in the high-suicide risk group the difference in voluntary admission rate between people diagnosed with personality disorder and other diagnosis is estimated at 0.37 versus 0.46 respectively (see Fig. 1a). The interaction effect suggests that when suicide risk increases, the probability of admission for patients with personality disorder increases more rapidly than for patients with no diagnosis of personality disorder. The interaction effect was more distinct in people who had been admitted involuntarily (for involuntary admissions model fit indices are higher). As we controlled for the risk of danger to others – which is strongly associated with involuntary admission – the coefficient for suicide risk is negligible in the model. When the level of suicide risk is moderate or high, the probability of involuntary admission for patients with a personality disorder is the same as that for patients with other disorders (see Fig. 1b).

The probability of (in)voluntary admission was also affected by other variables. An effect of motivation for treatment (main effect − 2.80, SE = .061; interaction effect − 0.257, SE = .042) indicated that involuntary admission was higher in patients with less motivation for treatment. Substance abuse did not change the effect of personality disorder on the association between suicide risk and the probability of admission. Family requests for admission increased the probability of voluntary admission, which rose in line with the level of suicide risk (main effect 1.59, SE = .036; interaction effect 0.105, SE = .026). Family support was also apparent in the assessment for involuntary admission but not in combination with suicide risk. Patients who had strong family involvement were less likely to be admitted to hospital, and were even less likely to be admitted when their suicide risk was higher (main effect − 0.114, SE = .032; interaction effect − 0.057, SE = .024). While these factors are important, they did not change the associations between level of suicide risk, personality disorder and (in)voluntary admission.

## Discussion

This study shows that suicidal patients diagnosed with a personality disorder are less likely to be voluntarily admitted to a psychiatric hospital by PES, as compared to patients not diagnosed with a personality disorder However, when suicide risk is higher, the personality disorder diagnosis becomes irrelevant in the case of an involuntary admission.

These findings partly confirm the speculation amongst clinicians that admission may be less effective and possibly harmful to people with a personality disorder. Apparently, clinicians working in the PES think that unless suicide risk is very high, suicidal patients with a personality disorder should not be admitted. We speculate that this might be due to fear for a deterioration of the clinical state of patients who have been admitted with a personality disorder.

Strong family support was also associated with a lower chance of both voluntary and involuntary admission, while family pressure on admission was associated with increased chances of (in)voluntary admission. A previous study showed similar results: when significant others requested admission, the probability of admission increased [27]. Family and friends also gave practical support and motivated patients to get better and adhere to their treatment, which decreased the probability of admission. The same study also showed that admission as the last available option is more likely to be unavoidable when family or other close relatives indicate that they can no longer provide help.

### Clinical significance and implications

When deciding on admission of a suicidal patient, PES professionals find themselves facing a recurrent dilemma: that admission might be harmful and increase suicidal behaviour – particularly in patients with a personality disorder – but that outpatient follow-up might not be safe enough. Data are lacking about both the effects on suicide risk of inpatient interventions [28]), as well as outpatient interventions such as Intensive Home Treatment (IHT). IHT can be seen as an alternative to admission, offers a multi-disciplinary approach and provides intensive community-based support and appropriate therapeutic interventions to patients and their families [29]. However, little is known about its effectiveness in prevention of suicide and a recent study suggests high suicide rates in IHT-patients [30], although causality remains unknown.

Another alternative to a voluntary admission to a psychiatric hospital may lie in respite houses that focus on a patient’s autonomy, empowerment and responsibility [31]. Patients can stay in such houses for a short while, accompanied by volunteers [31]. While this is promising, and may not lead to increased suicidal behaviour in patients with a personality disorder, there is as yet limited evidence of their effectiveness [32].

### Strengths and limitations

To our knowledge, this is the first study to describe the relationship between the level of suicide risk, personality disorder and psychiatric hospital admission. It nonetheless has some limitations. First, as personality disorders were diagnosed on the basis not of structured interviews, but on information gathered during the assessment by PES, some diagnoses may have been missing or incorrect. Therefore we grouped the various types of personality disorder together. Second, the assessment of suicide risk was based on one item of the SPI, and also not on a structured interview. Given the nature and pressure of working in the PES, however, using structured interviews is difficult. Third, as all data were collected in clinical practice, they were vulnerable to errors or missing data in some variables (ethnicity, age).

## Conclusions

After controlling for sociodemographic, clinical factors, psychiatric diagnoses and environmental factors, we intended this study to assess the association between level of suicidality and risk of voluntary or involuntary admission in patients presenting at the mobile PES with or without a personality disorder. We found that, independently of the level of suicide risk, suicidal patients diagnosed with a personality disorder were less likely to be admitted voluntarily than those without such a diagnosis. In involuntary admitted patients, however, personality disorder affected the probability of admission only in those whose risk of suicide was low Longitudinal studies are needed to better understand the associations between (in)voluntary admission and the course of suicidality in personality disorder patients.

## Abbreviations

PES: Psychiatric Emergency Service
SPI: Severity of Psychiatric Illness rating scale
